# A two-dimensional genome scan for rheumatoid arthritis susceptibility loci

**DOI:** 10.1186/1753-6561-1-s1-s63

**Published:** 2007-12-18

**Authors:** Jordana Tzenova Bell

**Affiliations:** 1 Wellcome Trust Centre for Human Genetics, University of Oxford, Oxford, OX3 7BN, UK

## Abstract

We performed a genome-wide search for pairs of susceptibility loci that jointly contribute to rheumatoid arthritis in families recruited by the North American Rheumatoid Arthritis Consortium. A complete two-dimensional (2D) non-parametric linkage scan was carried out using 380 autosomal microsatellite markers in 511 families. At each 2D peak we obtained the most likely underlying genetic model explaining the two-locus effects, defining epistasis as a departure from an additive or a multiplicative two-locus penetrance function. The highest peak in the surface identified an epistatic interaction between loci 6p21 and 16p12 (two-locus lod score = 18.02, epistasis *P *< 0.012). Significant and suggestive two-locus effects were also obtained for region 6p21 in combination with loci 18q21, 8p23, 1q41, and 6p22, while the highest 2D peaks excluding region 6p21 were observed at locus pairs 8p23-18q21 and 1p21-18q21. The 2D peaks were further examined using combined microsatellite and single-nucleotide polymorphism (SNP) marker genotypes in 744 families. The two-locus evidence for linkage increased for region pairs 6p21-18q12, 6p21-16p12, 6p21-8p23, 1q41-6p21, and 6p21-6p22, but decreased for pairs of regions that did not include locus 6p21. In conclusion, we obtained evidence for multi-locus interactions in rheumatoid arthritis that are mediated by the major susceptibility locus at 6p21.

## Background

Multiple loci are likely to influence susceptibility to rheumatoid arthritis (RA). Genome-wide scans for multiple interacting loci have been performed in model organisms [1] and more recently for complex human traits [2,3]. The hypothesis that genetic interactions contribute to RA has recently been examined using linkage analysis of selected regions [4]. However, a systematic genome-wide search for pair-wise interactions in RA has not yet been performed. The aim of this study was to carry out a genome-wide search for pairs of loci that jointly contribute to RA under two-locus genetic models that include epistasis. To achieve this, we performed a two-dimensional (2D) non-parametric linkage scan in sibling pairs affected with RA from the families in the North American Rheumatoid Arthritis Consortium (NARAC) collection. We detected a genome-wide significant epistatic interaction between loci 6p21 and 16p12, as well as several other pairs of loci that contribute to RA jointly and include locus 6p21.

## Methods

The genotyped sample of families provided by NARAC to the Genetic Analysis Workshop 15 consisted of 757 families (8017 individuals), in which at least one individual per family was genotyped [5,6]. We initially examined evidence for two-locus linkage using 380 autosomal microsatellite markers in 511 NARAC families, with 627 affected full-sib pairs (ASP), 29 affected maternal half-sib pairs (AMHSP), and 2 affected paternal half-sib pairs (APHSP). The peaks in the 2D surface were also tested for two-locus evidence for linkage using all of the available genotype data in the NARAC collection. There were 744 families (with 911 ASP, 43 AMHSP, and 2 APHSP) with genotypes available for either the autosomal microsatellite markers (380 markers), or for the autosomal single-nucleotide polymorphism (SNP) markers (5407 markers), or for all microsatellite and SNP autosomal markers (5787 markers). Marker order was based on build 18 of the human genome and genetic distances were obtained from the Rutgers map [7]. Where genetic distances were unavailable, we used physical distances to linearly interpolate the corresponding Rutgers cM location, and in cases where markers were located beyond the ends of the Rutgers map, we assumed that 1 cM = 1 Mb. In the lod-score calculations we used Haldane map units, founder allele frequencies, and if SNP markers were included in the analysis, we clustered markers using a linkage disequilibrium threshold of *r*^2 ^> 0.1.

Non-parametric linkage analysis was carried out initially using the single-locus maximum lod score (MLS) test statistic [8]. Two-locus non-parametric linkage analysis was performed using the two-locus extension of the MLS [9,10] implemented in Merloc [3]. Merloc uses likelihood estimates from Merlin [11] to estimate the joint two-locus allele sharing probabilities, which are then used in the calculation of the two-locus MLS using numerical maximization. The two-locus MLS under the most general two-locus model (GEN) is a function of the eight variance components at the two loci – the additive and dominance variances at locus 1 (V_A1 _and V_D1_) and 2 (V_A2 _and V_D2_), and the four epistatic variances (V_A1A2_, V_A1D2_, V_D1A2_, and V_D1D2_). Different genetic models can be fitted to the data by restricting the number of free variance components, for example, two-locus additive (ADD) or multiplicative (MUL) models and single-locus (SL) models are nested within the general epistatic model.

In the variance-component framework, epistasis can be defined as a departure from an additive or a multiplicative two-locus penetrance function. To assess the evidence for epistasis as a departure from additivity, the two-locus MLS under the general model may be compared to the MLS under the nested additive model with no interaction terms (GEN-ADD). Alternatively, a test for epistasis would compare the MLS under the general model with that under the multiplicative model (GEN-MUL). The MLS under a two-locus epsilon-epistatic model [3] (where a single parameter, *ε*, captures the degree of epistasis) and the maximum-likelihood estimate of *ε *can also be used to indicate the degree of epistasis (with *ε *= 0 corresponding to an additive model, *ε *= 1 to the multiplicative model, and *ε *> 10^3 ^to an extreme epistatic model).

We used previously published [3] significance thresholds to assess the significance of our findings of two-locus linkage compared to a null model in which neither locus affects the trait, where GEN = 5.85 corresponds to 2D genome-wide type I error rate of 0.05, and GEN = 4.30 corresponds to suggestive evidence for two-locus linkage. To assess the significance of the two-locus linkage results for pairs of loci that included 6p21, we performed two-locus simulations of chromosome pairs 6–16, 6–18, 6–8, and 6–1, by keeping chromosome 6 fixed (real data) against 1000 replicates of the simulated second chromosome (null effect). To estimate the point-wise significance of our findings of epistasis defined either as a departure from additivity or multiplicativity, we simulated two fully informative markers under the null two-locus model of interest (ADD for GEN-ADD and MUL for GEN-MUL). This was achieved by sampling from the observed two-locus allele sharing distribution under the null two-locus model (ADD or MUL) during the linkage analysis of our actual data. This procedure was performed at each 2D coordinate of interest and 100,000 replicates were used to obtain the GEN-ADD or GEN-MUL thresholds at that coordinate. A similar approach was applied to assess the significance of a secondary locus at 6p22. We generated 100,000 simulates of fully-informative markers, by sampling from the observed 6p21 single-locus allele sharing distribution, and subsequently we analyzed the replicates under the GEN model.

## Results

**Figure 1:**
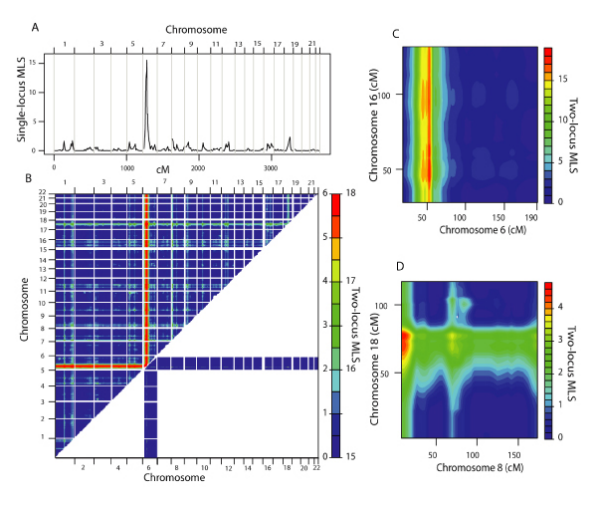
**Genome-wide linkage analysis of the microsatellite marker data**. A, Single-locus linkage results. B, Two-locus genome scan using the general two-locus model MLS. Above the diagonal are genome-wide results on a two-locus MLS scale of 0–6, and below the diagonal are chromosome 6 results on a two-locus MLS scale of 15–18. Highest fine-grid two-locus peaks in the 2D MLS surface, including (C) and excluding (D) chromosome 6.

Single-locus (SL) linkage analysis of the microsatellite genome-scan data in 511 NARAC families (Figure 1A) indicated a major locus on 6p21 (SL MLS = 15.55), and several loci with suggestive evidence for linkage (SL MLS > 1.5) at 1p21 (SL MLS = 1.67), 1q42 (SL MLS = 1.70), 5p15 (SL MLS = 1.51), 8p23 (SL MLS = 2.00), 12q15 (SL MLS = 1.51), and 18q21 (SL MLS = 2.3).

We performed a 2D linkage scan by computing the two-locus general model MLS at each marker-pair grid coordinate in the genome (Figure 1B). For each 2D peak we examined two-locus genetic models, starting with a general model that fits a wide range of epistatic models, and then restricted the number of free parameters in a stepwise manner to estimate the model that best fits the interaction (Table 1). To assess the significance of our findings, we initially used previously published simulation thresholds that assumed a null effect at both loci. The genome-wide significant and suggestive results using these simulations comprised all peaks including region 6p21 and two peaks (1p21-18q21 and 8p23-18q21) that excluded region 6p21.

**Table 1:** Highest two-locus peaks on the 2D surface

			Two-locus results^a^					
			
Locus 1	*Θ*	Locus 2	GEN (microsatellite)	GEN-ADD *P*-value	GEN-MUL *P-*value	ε^b^	GEN^c ^(microsatellite and SNP)	1-Lod unit support intervals (cM)^d^
Entire 2D surface								
6p21	0.5	16p12	18.02	**0.011**	**0.011**	10^3^	21.86	(51–55); (37–59)
6p21	0.5	18q21	17.90	0.254	0.937	0.66	23.04	(51–55); (52–85)
6p21	0.5	8p23	17.85	**0.036**	0.413	8.12	22.27	(51–55); (0–26)
1q42	0.5	6p21	17.44	0.642	0.083	0	21.58	(221–270); (51–55)
6p22	0.11	6p21	17.44	0.999	**0.009**	0	21.5	(31–47); (51–58)
2D surface excluding 6p21 interactions								
8p23	0.5	18q21	4.60	0.631	0.587	0	3.24	(0–26); (52–85)
1p21	0.5	18q21	4.33	0.114	0.410	53	3.42	(126–153); (26–85)

^a^Two-locus general model MLS obtained in 511 families with microsatellite genotypes alone. Empirical *P*-values assessing the significance of the difference in two-locus MLS under the general and additive models, and the general and multiplicative models. Significant deviations from the null two-locus model (ADD or MUL) are shown in bold. Empirical *P*-values were also obtained assuming a null effect at the two loci using published thresholds [3], and for pairs of loci excluding 6p21 these were estimated at *P *= 0.34 for 8p23-18q21, and *P *= 0.53 for 1p21-18q21.

^b^Maximum-likelihood estimate of epsilon.

^c^Two-locus general model MLS in 744 families using combined microsatellite and SNP data.

^d^1-Lod unit support intervals (to nearest Rutgers Kosambi cM) obtained using the GEN statistic from the microsatellite data set.

The highest two-locus general model MLS across the genome was obtained between two loci on 6p21 and 16p12 (Figure 1C; two-locus MLS = 18.02, *P *< 0.001) and the most likely model describing this interaction was a model of extreme epistasis (GEN-ADD *P *= 0.011, GEN-MUL *P *= 0.011, *ε *≥ 10^3^). Because the 2D peaks involving 6p21 always surpassed the genome-wide significance threshold of 5.85 (which assumes a null effect at either locus), we also assessed the significance of two-locus results involving 6p21 using simulations of chromosome pairs in which the null model included a single-locus effect at 6p21 alone. The results from these analyses indicated significant (chromosome-wide) effects at regions 16p12 (*P *= 0.012), 18q21 (*P *= 0.045), and 8p23 (*P *= 0.05) and a suggestive effect at 1q42 (*P *= 0.12), which was independent of 6p21. The two-locus effects observed at locus pairs 6p21-18q21, 6p21-8p23, 6p21-1q42, and 6p21-6p22 were best explained by the additive or multiplicative two-locus models (see Table 1). When 6p21 was excluded from the 2D surface, the highest two-locus MLS occurred between regions 8p23 and 18q21 (two-locus MLS = 4.6, *P *= 0.34) and the most likely underlying genetic model describing these effects was an additive two-locus model (Figure 1D). To attempt to refine the localization of the susceptibility regions, we also estimated 1-lod unit support intervals from the two-locus results (Table 1).

The peaks in the microsatellite 2D scan were also examined for two-locus evidence for linkage using all of the available genotype data in the NARAC collection. The results (Table 1) indicate that the evidence for linkage at the 2D peaks involving 6p21 increases, in particular for locus pair 6p21-18q21, however, the evidence for linkage involving loci 8p23-18q21 and 1p21-18q21 decreases.

**Figure 2:**
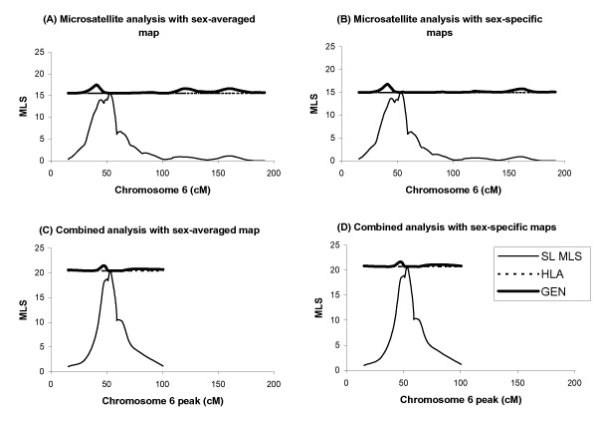
**Two-locus fine-scale linkage analysis of chromosome 6**. Single-locus (SL MLS) and two-locus (GEN) analyses of chromosome 6 were performed assuming a disease locus at D6S1629 (HLA denotes the single-locus MLS score at D6S1629). Results for the microsatellite markers using sex-averaged (A) and sex-specific (B) genetic maps, and the combined microsatellite and SNP markers using sex-averaged (C) and sex-specific (D) genetic maps.

We also examined the evidence for two linked loci on chromosome 6 in more detail, using sex-averaged and sex-specific maps (Figure 2). Assuming that a major RA locus is present at 6p21, the evidence for a secondary RA locus on chromosome 6 is highest at marker D6S2439 under an additive two-locus model. The MLS at D6S2439, independent of D6S1629, ranges between 0.93 and 1.9, depending on the inclusion of the SNP data in the analysis and whether sex-averaged or sex-specific distances are used. The evidence for linkage at D6S2439 is highest in the microsatellite analyses using the sex-averaged map, 1.9 (*P *= 0.004), and is lowest in the analyses of the combined data under sex-specific maps, 0.93 (*P *= 0.048).

## Discussion and conclusion

We performed a genome-wide search for pair-wise interactions that contribute to RA susceptibility in the NARAC family collection. The highest peak on the 2D surface involved an epistatic interaction between two loci on 6p21 and 16p12. We also detected pairs of loci that jointly contribute to RA under two-locus additive and multiplicative models, 6p21-18q21, 6p21-8p23, and 6p21-1q41. Suggestive evidence for a secondary gene on 6p22, independent of the major locus on 6p21, was also obtained, but addition of SNP genotypes and use of more precise sex-specific maps in the two-locus analyses of chromosome 6 reduced the evidence for linkage at 6p22, indicating that these results should be interpreted with caution. Our findings are consistent with previous interaction analyses of genetic interactions in RA [4]. John et al. [4] examined evidence for epistasis among selected RA susceptibility regions in the families used in this study and additional data, defining interaction as a departure from a multiplicative two-locus model. The two-locus results obtained at locus pair 6p21-16p12 coincide exactly in the two studies, however, although we observe a two-locus peak at region pair 6p21-6q16 (Figure 2), the magnitude of the peak does not attain significance, which might be due to differences in genetic maps and slight changes in the data structure between the two studies.

Multi-locus linkage analysis methods have been developed and applied to complex human traits. Such methods are useful in detecting novel loci that contribute to the trait susceptibility only through their genetic interactions, and in establishing the type of interaction among susceptibility loci. The approach used in this study can examine entire genomes for potential pair-wise or higher-order interactions, however, interpreting the genome-wide significance of the findings is challenging, in particular when there is strong single-locus evidence for linkage. All of the regions involved in the 2D peaks in our analyses have at least suggestive single-locus effects. Therefore, the results of this study are more useful for elucidating the nature of the interactions between previously identified RA susceptibility loci, rather than in identifying novel loci for RA susceptibility. The two-locus findings are also useful in potentially refining the susceptibility regions, by yielding narrower 1-lod unit support intervals.

Our primary results were based on analyses of the microsatellite genotype data alone, while SNP genotypes were added to confirm the effects observed at the two-locus peaks. The inclusion of SNP genotypes in the 2D scan of the peaks allowed for more informative analyses, with more confidence in the two-locus effects observed at the 2D peaks. A follow-up to this study would involve a complete 2D genome-wide analysis of the combined SNP and microsatellite data in the NARAC families. These analyses prove computationally prohibitive at present.

The analyses of the NARAC data indicated that if genetic interactions contribute to RA, they are most likely mediated by the major locus at 6p21. This study was aimed at searching for pair-wise interactions in RA, but higher dimension interactions may also exist and should be examined in future analyses.

## Competing interests

The author(s) declare that they have no competing interests.
